# Assessing Treatment-Resistant Posttraumatic Stress Disorder: The Emory Treatment Resistance Interview for PTSD (E-TRIP)

**DOI:** 10.3390/bs4040511

**Published:** 2014-12-08

**Authors:** Boadie W. Dunlop, Joanna L. Kaye, Cole Youngner, Barbara Rothbaum

**Affiliations:** 1 Department of Psychiatry and Behavioral Sciences, Emory University School of Medicine, 12 Executive Park Drive, 3rd Floor, Atlanta, GA 30329, USA; E-Mails: jlk399@drexel.edu (J.K.); brothba@emory.edu (B.R.); 2 Department of Psychology, Drexel University, 3141 Chestnut Street, Philadelphia, PA 19104, USA; 3 Rollins School of Public Health, Emory University, 1518 Clifton Road, Atlanta, GA 30322, USA; E-Mail: cyoungn@emory.edu

**Keywords:** evidence-based medicine, treatment outcomes, antidepressive agents, psychotherapy, psychiatric status rating scales, treatment failure

## Abstract

Patients with posttraumatic stress disorder (PTSD) who fail to respond to established treatments are at risk for chronic disability and distress. Although treatment-resistant PTSD (TR-PTSD) is a common clinical problem, there is currently no standard method for evaluating previous treatment outcomes. Development of a tool that could quantify the degree of resistance to previously provided treatments would inform research in patients with PTSD. We conducted a systematic review of PTSD treatment trials to identify medication and psychotherapy interventions proven to be efficacious for PTSD. We then developed a semi-structured clinician interview called the Emory Treatment Resistance Interview for PTSD (E-TRIP). The E-TRIP includes clinician-administered questions to assess the adequacy and benefit derived from past treatment trials. For each adequately delivered treatment to which the patient failed to respond, a score is assigned depending on the strength of evidence supporting the treatment’s efficacy. The E-TRIP provides a comprehensive assessment of prior PTSD treatments that should prove valuable for researchers studying TR-PTSD and evaluating the efficacy of new treatments for patients with PTSD. The E-TRIP is not intended to guide treatment; rather, the tool quantifies the level of treatment resistance in patients with PTSD in order to standardize TR-PTSD in the research domain.

## 1. Introduction

Several evidence-based psychotherapies and medications are available to treat posttraumatic stress disorder (PTSD). However, many patients do not respond to these treatments, placing them at risk for developing a chronic course of illness and poor long-term outcomes [1].

Despite the clinical significance of treatment resistant PTSD (TR-PTSD), there is currently no standard tool to identify patients as treatment resistant, and there is no agreement on what level of prior treatment should constitute TR-PTSD [2]. While there are several published guidelines to help clinicians make treatment choices for patients with PTSD, each guideline differentially weights certain aspects of study design and outcomes when reviewing clinical trials, which has resulted in substantial variability about treatment efficacy across guidelines [3]. The lack of agreement about treatment efficacy presents a challenge for developing a measure of TR-PTSD because defining treatment resistance requires a single standard for efficacy. Moreover, the guidelines were intended to help guide treatment decisions for patients with PTSD, rather than quantify the level of treatment resistance. Thus, there is a need for a TR-PTSD measure that could be incorporated into clinical research to characterize patients’ previous treatment outcomes. 

We conducted a systematic review of the evidence to determine which treatments have sufficient evidence of efficacy to warrant inclusion in a measure of TR-PTSD. Herein, we describe the development of the Emory Treatment Resistance Interview for PTSD (E-TRIP), a semi-structured interview designed to quantify TR-PTSD.

## 2. Methods

### 2.1. Systematic Review 

We conducted a systematic review of the literature by searching titles for the words “PTSD” and its variants (“posttraumatic stress disorder”, “post traumatic stress disorder”, and “post-traumatic stress disorder”) and “treatment” or “randomized trial” or “randomized study” into comprehensive databases (PsycInfo, PubMed, and PILOTS) through 15 December 2013. We included studies designed to treat patients with PTSD and other comorbid disorders (e.g., substance abuse) or designed to decrease sleep-related symptoms of PTSD if they analyzed overall PTSD symptom change. In order to locate studies that did not return in our database searches, we also examined studies reviewed in *Effective Treatments for PTSD: Practice Guidelines from the ISTSS* [4]. 

Table 1 presents our criteria for determining treatment efficacy. In selecting these criteria, we erred on the side of setting the bar higher rather than lower in order to avoid labeling a patient as treatment-resistant if it is not clear they received an adequate trial of a proven intervention. We required trials to demonstrate statistical superiority of a treatment using the trial’s intent-to-treat (ITT) sample, as ITT analyses provide the most unbiased results [5]. We requested ITT analyses from authors of trials whose published trials reported only treatment-completer results or who did not indicate which sample was analyzed. We set a minimum sample size per arm of 16 subjects. We derived this sample size for 1:1 parallel group randomization studies by applying a Student’s t-test using a ten-point difference in total Clinician Administered PTSD Scale (CAPS) score between treatment arms (the value considered to reflect a clinically significant improvement) [6,7], a standard deviation of ten, and a two-sided alpha < 0.05. We used similar assumptions for calculating minimum sample sizes for other study designs (e.g., cross-over studies, imbalanced randomization).

**Table 1:** Study Inclusion Criteria.

Adults (age ≥ 17)
Current PTSD diagnosis
Published in English in peer-reviewed journal
PTSD outcome measure with high reliability and validity - Clinician-Administered PTSD Scale [6] - Davidson Trauma Scale [8] - Impact of Event Scale [9] - PTSD Checklist [10] - Posttraumatic Diagnostic Scale [11] - PTSD Symptom Scale [12]
Randomized treatment allocation
If evaluating a single modality, includes a minimal control group (e.g., placebo, wait-list, or psychotherapy control condition)
If an augmentation treatment study (in which an experimental intervention was added to an established treatment), includes a control condition arm that did not employ the experimental intervention
Treatment group improves significantly more than the control group on the PTSD outcome measure at a 2-sided alpha < 0.05 for the intent-to-treat sample
Adequate sample size (see text)

### 2.2. E-TRIP Development

For the E-TRIP, we determined that a continuous measure of treatment resistance would provide greater value than a tool that assigns categorical stages. Consistent with the continuous measures used to define treatment-resistant depression (TRD) [13,14], we assigned point values reflecting failure to respond to specific treatments. We identified nine key areas of uncertainty to resolve in assessing TR-PTSD, described below.

#### 2.2.1. Defining What Constitutes a Demonstrably Efficacious Treatment for PTSD 

Consistent with the majority of the treatment guidelines, we set the definition of an efficacious treatment as an intervention with at least one published positive RCT. 

#### 2.2.2. Differential Weighting of Treatments

Some measures of TRD apply greater weights to specific treatments, such as electroconvulsive therapy [13]. For PTSD, no single treatment is proven superior in the same manner that ECT is for MDD. However, replication of efficacy offers greater confidence that the observed benefit does not arise from chance or from unique characteristics of the study sample [15]. Differential weights are also justified for monotherapies, which target the entire illness, *versus* augmentation or combination treatments which typically aim to improve aspects of the illness not responsive to a monotherapy.

For the purposes of the E-TRIP, we define an “augmentation” medication and a “combination” psychotherapy as a treatment that is not an established monotherapy but which is added to an established monotherapy medication or psychotherapy in order to improve response. 

We assigned the following weights for treatments to which the patient did not respond:

- 3 points: Treatments with demonstrated efficacy in multiple (>1) RCTs.
- 2 points: Treatments with a single positive RCT.
- 1 point: Augmentation medications or combination psychotherapies with at least one positive RCT. For these treatments there are no additional weighting added for replications of efficacy.
- 0 points: Treatments without a positive RCT demonstrating its efficacy.

Agents proven efficacious as monotherapies are not scored as augmentation agents when they are given either concurrently or sequentially with another proven monotherapy; rather, both monotherapies are scored individually. For example, a patient who fails to benefit from concomitantly-delivered adequate treatments of exposure therapy and a selective serotonin reuptake inhibitor (SSRI) would score six points, because both treatments have three points for evidence of efficacy. 

#### 2.2.3. Defining the Minimum Levels of Dose and Duration that Constitute Adequate Exposure to a Treatment

Determining adequacy of prior treatments involves a trade-off between requiring a definitive exposure to a proven adequate treatment *versus* the need for criteria that reflect real-world patterns of practice. To ensure the E-TRIP tool can be practical while maintaining sufficient rigor, we defined minimum dose and duration aspects of treatment by striking a balance between “optimal” *versus* “routine” treatment delivery.

For medication, we determined that for monotherapies, a minimum of eight weeks of treatment at a minimum effective dose is required to evaluate efficacy, which is consistent with other reviews [2,16]. By using the eight-week cut-off, we are not asserting that further medication benefits do not accrue beyond that point; indeed, sustained treatment is associated with continued gains and progression to remission [17]. Rather, the evidence indicates eight weeks on a medication is an adequate period to determine whether a 30% improvement is likely to occur. The only exception to the eight-week minimum duration is for eszopiclone when used as an augmentation agent; for this medication, a minimum duration of ≥3 weeks is required, based on the duration of the RCT supporting its efficacy [18].

In recognition that many patients in clinical care are not pushed to maximally-tolerated doses, we chose to consider the medication’s minimum effective dose (determined from the minimum effective doses used in efficacy trials) as the threshold to define an adequate trial. For some effective medications, the minimum necessary dose is unclear; for example, prazosin has a very wide dosing range, and the range differs for men and women [19,20]. In these cases, we used our best judgment, based on the literature, to select a minimally effective dose. For antidepressants that have not been studied in PTSD, the E-TRIP uses the minimum effective dose employed for major depressive disorder, based on the observation that for the SSRIs and venlafaxine, the minimally effective dose for PTSD and for MDD are equivalent [21,22]. For other classes of medications that have not demonstrated efficacy in PTSD, we do not list a minimum effective dose. We note that investigators wanting to set a more definitive standard of treatment failure with prior drug treatments could substitute higher minimum dose thresholds.

For psychotherapy treatments, we determined the minimum number of sessions by reviewing the number used in the clinical trials demonstrating efficacy by our inclusion criteria. For exposure-based treatments, we identified only two analyses reporting response rates by session number, which found that attending six or more sessions was associated with greater improvement [23,24]. We decided to require a minimum of six sessions of therapy to constitute an adequate course of treatment. While some patients require a longer duration of therapy to achieve maximal response, many patients who complete six sessions of treatment experience a significant level of improvement in the frequency and severity of anxious and depressive symptoms as well as functional status [23].

#### 2.2.4. Definition of Treatment Outcome

Mood and anxiety disorder treatment research generally recognizes two categories of positive outcomes: response and remission. We determined that requiring remission to be classified as efficacious sets the bar too high for a treatment to show benefit for PTSD. Therefore, we opted to use response as the standard for an effective treatment. Several symptom rating scales have been used to define response in PTSD; the most commonly used definitions are a ≥ 30% reduction in the CAPS or a score of at least “much improved” (score ≤ 2) on the Clinical Global Impressions-Improvement scale [25,26]. In contrast, psychotherapy studies often use the outcome of “good end-state functioning,” typically a conglomeration of several measures [27]. Because the great majority of treatment studies define response based on a symptom severity scale, the E-TRIP assesses response to treatment on the basis of the patient’s reported change in the intensity, duration, and frequency of symptoms and associated behavioral impairments. 

Subjective recall of treatment benefit is susceptible to inaccuracy [28], but is the basis for commonly used tools in TRD, such as the Antidepressant Treatment Response Questionnaire [29]. Many patients find these self-report treatment forms difficult to complete due to the complexity of the information requested. Thus, for the E-TRIP, we require the interviewer to determine the degree of benefit derived from previous treatments through the use of semi-structured questions regarding symptom change during treatment. We selected an overall symptom improvement level of 30% as the threshold for response based on the commonly used clinical trial definition of response of ≥30% reduction in total CAPS score [25,30].

#### 2.2.5. Lifetime Treatment Response *versus* Most Recent Episode Response

Though the severity of PTSD may wax and wane with life stressors, the illness is not considered to be an episodic disorder [31]. Because there are no established means for defining a current episode or exacerbation of PTSD, the E-TRIP assesses each treatment across the lifespan. 

#### 2.2.6. Distinguishing between Intolerance and Non-Response

Some patients may be unable to sufficiently tolerate a medication or psychotherapy to allow an adequate duration or intensity of treatment. Although intolerance to a medication may present differently than intolerance to a psychotherapy, in both cases adjusting aspects of the treatment delivery (e.g., dosing titration with medication, or degree of emphasis on education or relaxation during psychotherapy) may enable a patient to tolerate a rechallenge with a previously intolerable treatment. In the TRD literature, intolerance of treatment is distinct from a treatment being unable to provide benefit for the disorder. Similarly, E-TRIP treatments discontinued or under-dosed due to intolerance are not considered adequately delivered and do not count toward the E-TRIP score; including points toward the treatment resistance score for intolerance of a treatment would reduce the specificity of TR-PTSD.

#### 2.2.7. Classification of Medications Individually or by Class

Significant uncertainty exists around the degree to which treatments of the same class or type should be considered equivalent. To the extent they are equivalent, scoring each trial of a related treatment may exaggerate the true level of treatment resistance. Because the SSRIs share a primary mechanism of action and there is strong evidence for the efficacy of multiple SSRIs, we grouped all SSRIs together as a class of treatment. In contrast, due to the lack of consistent evidence of efficacy within other drug classes, the E-TRIP assesses all other medications individually based on their RCT efficacy data. 

Because there are several SSRIs, patients could potentially receive a high treatment resistance score without exposure to any other form of treatment. Thus, the patient’s E-TRIP score counts a maximum of two SSRI treatment failures. Patients who have failed three or more SSRI treatments would count as having only two SSRI treatment failures; thus the maximum points a patient could receive towards resistance to treatment with SSRIs is six points. This compromise recognizes that some patients may respond preferentially to one SSRI over another but limits the treatment resistance score that can derive from a single class of treatment. Unlike the limit we assigned to SSRI-group scoring, we did not limit the number of points that could be scored for patients who completed multiple courses within a class of psychotherapy (e.g., trauma-focused CBT). This decision was based on the uncertainty that all trauma-focused therapies engage the same mechanisms of recovery. Mitigating this concern, we note that in our clinical experience very few patients with PTSD have completed more than two forms of trauma-focused CBT. 

#### 2.2.8. Clinical Features

Some measures of TRD incorporate chronicity or severity of illness in determining a treatment resistance score [14], suggesting factors other than treatment outcomes per se may predict future treatment resistance. In PTSD, a variety of clinical features have predicted treatment outcome in individual studies, including time since trauma, number of lifetime traumas, injury at time of trauma, severity of PTSD, and presence of childhood trauma [32]. However, replication of these predictors has been inconsistent, and which of these putative predictors has the greatest influence on treatment outcomes remains unclear. We decided not to include non-treatment clinical variables in the E-TRIP due to the uncertainty around which clinical variables are most salient. 

#### 2.2.9. Assessing Adherence to Prior Treatments

Poor adherence to medication treatment is common among patients with PTSD and other anxiety disorders [33,34]. Clinical trials often consider 80% adherence to a drug regimen the minimal adherence needed to ensure adequate treatment exposure [35]. As part of the E-TRIP, the interviewer asks structured questions about adherence and duration of treatment. Whenever possible, researchers should independently verify patient responses using pharmacy records. For medication trials, we use an adherence threshold of six days per week to reflect 80% adherence to daily dosing regimens.

## 3. Results

### 3.1. Review of Available PTSD Treatment Outcome Literature

**Figure 1:**
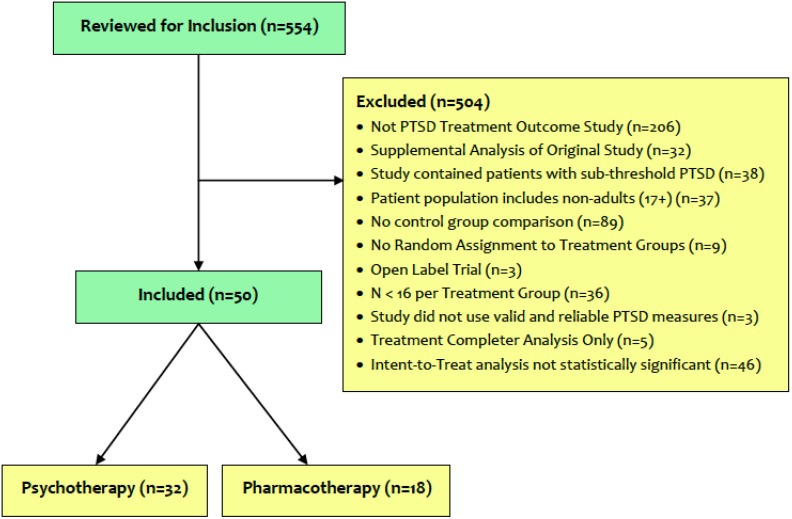
CONSORT diagram of eligible studies for inclusion in the E-TRIP.

Our searches identified 554 potentially eligible studies for inclusion. Reasons for study exclusion are presented in Figure 1. Of the remaining 50 eligible studies, 32 evaluated psychotherapeutic interventions, and 18 evaluated medication treatments. The pharmacotherapy treatment studies that met our criteria for efficacy are presented in Table 2. The psychotherapy treatment studies that met our criteria for efficacy are presented in Table 3. 

**Table 2:** Pharmacotherapy treatments scored by the E-TRIP.

Treatment	Points	Supporting Studies
**SSRIs**		
Citalopram	3	
Escitalopram	3	
Fluoxetine	3	Connor *et al.* [36], Martenyi *et al.* [37]
Fluvoxamine	3	
Paroxetine	3	Marshall *et al.* [38,39], Tucker *et al.* [40], Schneier *et al.* [41]
Sertraline	3	Brady *et al.* [42], Davidson *et al.* [21], Panahi *et al.* [43]
Vilazodone	3	
**SNRIs**		
Venlafaxine	3	Davidson *et al.* [22,44]
**TCAs**		
Imipramine	2	Kosten *et al.* [45]
**MAOIs**		
Phenelzine	2	Kosten *et al.* [45]
**Other Antidepressants**		
Nefazodone	2	Davis *et al.* [46]
**Atypical Antipsychotics**		
Risperidone	1	Bartzokis *et al.* [47]
**Sedatives**		
Eszopiclone	1	Pollack *et al.* [18]
**Other Medications**		
Prazosin	1	Raskind *et al.* [19,48]
Topiramate	1	Akuchekian and Amanant [49]

**Table 3:** Psychotherapy treatments scored by the E-TRIP.

Treatment	Points	Supporting Studies
**Trauma-Focused CBT**		
Prolonged Exposure (PE)	3	Cloitre *et al.* [50], Resick *et al.* [51], Foa *et al.* [52], Rothbaum *et al.* [53], Schnurr *et al.* [54], Mills *et al.* [55], Pacella *et al.* [56]
Cognitive Processing Therapy (CPT)	3	Resick *et al.* [51], Chard [57], Monson *et al.* [58], Forbes *et al.* [59]
Trauma-Focused Cognitive-Behavioral Therapy (TFCBT)	3	Ehlers *et al.* [60], Kubany *et al.* [61,62], Duffy *et al.* [63], Hollifield *et al.* [64], Cottraux *et al.* [65], Mueser *et al.* [66]
**Internet-based Therapies**		
Internet-Based Cognitive-Behavioral Therapy	3	Knaevelsrud and Maercker [67], Litz *et al.* [68], Spence *et al.* [69]
**Group Therapies**		
Group Interpersonal Therapy (IPT)	3	Krupnick *et al.* [70]
Cognitive-Behavioral Conjoint Therapy (CBCT)	2	Monson *et al.* [71]
**Complementary and Alternative Medicine Therapies**		
Mindfulness	2	Niles *et al.* [72]
Acupuncture	2	Hollifield *et al.* [64]
Healing Touch with Guided Imagery	2	Jain *et al.* [73]
**Other Therapies**		
Resiliency Intervention	2	Kent *et al.* [74]
Emotional Freedom Techniques (EFT)	2	Church *et al.* [75]
Mind-Body Bridging Program for sleep management	2	Nakamura *et al.* [76]
**Combination Therapies**		
Acupoint Stimulation added to Cognitive-Behavioral Therapy	1	Zhang *et al.* [77]

### 3.2. Using the E-TRIP

The E-TRIP form is presented in Supplementary 1 and a scoring example for the E-TRIP is presented in Supplementary 2. Administration of the E-TRIP begins with the patient completing the PTSD Medication Treatment Record and PTSD Psychotherapy Treatment Record to indicate which treatments the patient has previously received. The interviewer then collects these records and starts the interview by determining the onset of PTSD and primary symptoms affecting the patient. The interviewer then assesses the timing, dosage, duration, adherence, and response for each treatment trial indicated by the patient and any available clinical records and enters responses on the E-TRIP Treatment Records. Points are scored for each treatment with proven efficacy to which the patient failed to respond. Separate totals are compiled for medication treatment failures, psychotherapy treatment failures, and all treatments combined. No points are given for failure to respond to treatments with no proven efficacy.

We administered the E-TRIP to 22 participants who were seeking to participate in a clinical trial for PTSD. The sample was comprised of 17 women and five men, with a mean age of 39.0 ± 11.8 years. The participants were 54.5% Caucasian, 36.4% African-American, and 9% other races, and 4.5% were Hispanic. The most common index trauma was sexual assault (45.5% of the sample), followed by military combat (22.7%) and non-sexual assault (9.1%). Education level of the sample was moderately high, with 91% attending at least some college and 41% achieving a Bachelor’s Degree or higher, but 68.2% of the sample had an annual income less than US $25,000 per annum. The time required to administer the E-TRIP ranged from one minute for treatment-naïve patients to 20 minutes for more extensively treated patients. Mean time to administer the instrument was 6.41 ± 5.47 minutes. 

## 4. Discussions

We conducted a review of the existing evidence base of treatments for PTSD in order to develop a measure of TR-PTSD, the E-TRIP. As a standardized method for assessing treatment resistance, the E-TRIP aims to increase researchers’ ability to compare sample characteristics across studies. Furthermore, the E-TRIP scores may be valuable for developing inclusion and exclusion criteria for clinical trials and for biological investigations into the nature of TR-PTSD. In clinical efficacy trials, E-TRIP scores could help stratify patients pre- or post-hoc in order to better understand treatment outcomes. 

Our review of the efficacy literature was not performed with the intent of developing a treatment guideline or recommendations for treatment. Nevertheless, our review did find similar areas of agreement with the major published guidelines as a whole [3], such as high-level support for trauma-focused psychotherapy, SSRIs and venlafaxine. Both our review and all the guidelines used RCTs to signify the highest level of evidence of efficacy, but the guidelines also considered other forms of evidence beyond RCTs in developing recommendations. 

In constructing the E-TRIP Treatment Records, we included many treatments for which failure to respond scores no points because many patients do not receive evidence-based treatments for PTSD. We expect that future trials conducted with treatments currently listed as having inadequate proof of efficacy may yield positive efficacy data, which will be incorporated through periodic revisions of the E-TRIP as efficacy data accrues. 

Broader use of the E-TRIP may significantly contribute to our understanding of TR-PTSD, though further work is required to confirm the validity and inter-rater reliability of the E-TRIP, including use of the instrument across sites and with a greater variety of patients. Treatment resistance is better considered as a spectrum phenomenon, rather than simply as a feature that is present or absent. Identifying patterns of treatment non-response using the E-TRIP may help identify patients who are more likely to benefit from certain interventions compared to others, thereby helping construct more personalized treatment algorithms. Thus, beyond the research applications described above, the E-TRIP may eventually find value in clinical practice. 

The primary limitation of the E-TRIP is that it may underestimate the level of treatment resistance by failing to allocate points for treatments that actually have efficacy but currently lack RCT data demonstrating benefit. We decided to adhere tightly to the published evidence base in defining efficacious treatments for two reasons. First, going beyond the RCTs introduces many subjective judgments about which reasonably-informed experts may disagree. Second, we wished to err on the conservative side in determining a patient’s level of treatment resistance. Overly broad inclusions for treatments would result in the E-TRIP scores having less specificity for TR-PTSD and thus introduce greater heterogeneity among patients identified as having TR-PTSD. Our decision to score both components of concurrently delivered proven monotherapies (e.g., SSRI with prolonged exposure) may risk inflating the treatment resistance score (because it reflects only one period of treatment). 

An additional limitation of the E-TRIP concerns the assumptions made in defining minimally adequate exposure and the points assigned for the various treatments. There is substantial uncertainty in the literature around minimum effective doses for certain medications and the minimum number of sessions of psychotherapy required before concluding a given treatment is ineffective. The educated assumptions we made in arriving at the values used in the E-TRIP will need to be tested through clinical trials using randomization to fixed doses of medication or a variable number of psychotherapy sessions. Similarly, our scoring system of two or three points for monotherapy failures and one point for an augmentation failure will need to be tested through studies in which this proposed scoring is compared against alternative scoring systems for their relative value in prospectively predicting treatment non-response. Our decision to limit the number of points score to two failed SSRI trials (as opposed to one or three SSRI trials) will also need prospective evaluation. 

## 5. Conclusions 

The E-TRIP is a semi-structured interview tool that will enhance researchers’ ability to quantify the degree of treatment resistance in patients with PTSD through a standardized assessment. With this measure, researchers may reduce heterogeneity between patients participating in clinical research studies, allowing for greater confidence in study results. As future, potentially more invasive treatments are developed for TR-PTSD, it will be important to document TR-PTSD in a consistent manner.

## Supplementary Files

Supplementary File 1

Supplementary File 2
