# Supplementary material for: Assessing Treatment-Resistant Posttraumatic Stress Disorder: The Emory Treatment Resistance Interview for PTSD (E-TRIP)
Source: Behav Sci (Basel). 2014 Dec 8;4(4):511–27. doi: 10.3390/bs4040511 (PMC4287702; doi:10.3390/bs4040511)
Supplement: Supplementary File 1 [file behavsci-04-00511-s001.pdf]

OVERVIEW

- Start the E-TRIP assessment by handing the **PTSD Medication Treatment Record** and **PTSD Psychotherapy Treatment Record** pages to the patient. The patient should indicate the treatments they have previously received by marking the check boxes next to the treatments. Advise the patient that their only action on the form is to check the boxes in the white (unshaded) areas; they should ignore the rest of the form (these areas can also be covered so as not to confuse patients completing the form).
- Medications are grouped by classes for ease of organization, and antipsychotics are included in "other agents" to avoid the possible misinterpretation that they may only be used for patients with psychotic symptoms. For each efficacious psychotherapy, a single sentence description is provided to help patients who may not have been told the specific name of the psychotherapy they received.
- After the patient has completed the Treatment Records, the interviewer collects them and administers the semi-structured interview.
- Questions in **Bold** font should be asked as written.
- Text within boxes provides instructions to the interviewer for how to proceed and how to mark the Treatment Record.
- Each shaded gray box (for instance, **Start Date** MM/YYYY) indicates with which column on the Treatment Record the instruction corresponds.
- The interview begins by assessing the onset of PTSD and primary symptoms; if this information is already known, these questions do not need to be asked, although the relevant information should be recorded. Identifying onset of PTSD is crucial for determining treatment resistance, because many patients who had episodes of anxiety or depression before experiencing a trauma may record on the treatment records that they had received specific treatments, though in fact those treatments were administered prior to the onset of PTSD and therefore should not contribute to the E-TRIP score.
- Next, the interviewer evaluates the response to the individual treatments. The interview has separate sections for medication and psychotherapy treatments. For each individual treatment for which the patient has checked the box, the interviewer proceeds through the interview, one treatment at a time, following the instructions and recording answers in the Treatment Record. For instance, if a patient had been treated with sertraline and venlafaxine, the interviewer would ask questions 3-5 in regards to sertraline, then return to question 3 and assess venlafaxine. This applies to psychotherapy treatments as well.

NOTE: For psychotherapy and medication treatments administered concomitantly, the key consideration is whether the patient responded while receiving the treatments. If so, no points for resistance will be scored. If the patient did not respond, then points should be scored for both treatments, just as if they had been administered separately.

PATIENT ID: DATE: INTERVIEWER:

E-TRIP PTSD MEDICATION TREATMENT RECORD

Have you ever taken a prescription medication for PTSD? ☐ No ☐ Yes

If yes, please review the list of medications below and check the box (☐) next to any you have taken in the past or are taking now.

DO NOT WRITE IN THE GRAY-SHADED AREAS

PATIENT SECTION			OFFICE USE ONLY									
**Generic name**	**Trade name**	**Check box if ever taken**	OFFICE USE ONLY	**Start date** MM/YYYY	**For PTSD?**	**Dose used**	**Minimum effective dose**	**Used to augment?**	**≥ 8 weeks?**	**≥6 days/ week?**	**If adequate trial, responded?**	**Points†**
**SSRIs**												
Citalopram	Celexa	☐			Y N		20 mg/d	Y N	Y N	Y N	Y N U	3
Escitalopram	Lexapro	☐			Y N		10 mg/d	Y N	Y N	Y N	Y N U	3
Fluoxetine	Prozac	☐			Y N		20 mg/d	Y N	Y N	Y N	Y N U	3
Fluvoxamine	Luvox	☐			Y N		50 mg/d	Y N	Y N	Y N	Y N U	3
Paroxetine	Paxil (Paxil CR)	☐			Y N		20 mg/d (25)	Y N	Y N	Y N	Y N U	3
Sertraline	Zoloft	☐			Y N		50 mg/d	Y N	Y N	Y N	Y N U	3
Vilazodone	Viibryd	☐			Y N		20 mg/d	Y N	Y N	Y N	Y N U	3
**SNRIs**												
Desvenlafaxine	Pristiq	☐			Y N		50 mg/d	Y N	Y N	Y N	Y N U	0
Duloxetine	Cymbalta	☐			Y N		40 mg/d	Y N	Y N	Y N	Y N U	0
Venlafaxine	Effexor	☐			Y N		75 mg/d	Y N	Y N	Y N	Y N U	3
**TCAs**												
Amitriptyline	Elavil	☐			Y N		150 mg/d	Y N	Y N	Y N	Y N U	0
Clomipramine	Anafranil	☐			Y N		150 mg/d	Y N	Y N	Y N	Y N U	0
Desipramine	Norpramin	☐			Y N		150 mg/d	Y N	Y N	Y N	Y N U	0
Doxepin	Sinequan	☐			Y N		150 mg/d	Y N	Y N	Y N	Y N U	0
Imipramine	Tofranil	☐			Y N		150 mg/d	Y N	Y N	Y N	Y N U	2
Nortriptyline	Pamelor	☐			Y N		75 mg/d	Y N	Y N	Y N	Y N U	0
**MAOIs**												
Phenelzine	Nardil	☐			Y N		45 mg/d	Y N	Y N	Y N	Y N U	2
Selegiline	Emsam	☐			Y N		6 mg/24 hrs	Y N	Y N	Y N	Y N U	0
Tranylcypromine	Parnate	☐			Y N		30 mg/d	Y N	Y N	Y N	Y N U	0
**OTHER ANTIDEPRESSANTS**												
Bupropion	Wellbutrin	☐			Y N		300 mg/d	Y N	Y N	Y N	Y N U	0
Mirtazapine	Remeron	☐			Y N		30 mg/d	Y N	Y N	Y N	Y N U	0
Nefazodone	Serzone	☐			Y N		300 mg/d	Y N	Y N	Y N	Y N U	2
Trazodone	Desyrel	☐			Y N		300 mg/d	Y N	Y N	Y N	Y N U	0

TURN TO NEXT PAGE

E-TRIP PTSD MEDICATION TREATMENT RECORD

PATIENT SECTION			OFFICE USE ONLY								
**Generic name**	**Trade name**	**Check box if ever taken**	**Start date** MM/YYYY	**For PTSD?**	**Dose used**	**Minimum effective dose**	**Used to augment?**	**≥8 weeks?**	**≥6 days/ week?**	**If adequate trial, responded? (Y, N, U)**	**Points†**
**BENZODIAZEPINES/SEDATIVES**											
Clonazepam	Klonopin	☐		Y N		n/a	Y N	Y N	Y N	Y N U	0
Lorazepam	Ativan	☐		Y N		n/a	Y N	Y N	Y N	Y N U	0
Alprazolam	Xanax	☐		Y N		n/a	Y N	Y N	Y N	Y N U	0
Diazepam	Valium	☐		Y N		n/a	Y N	Y N	Y N	Y N U	0
Eszopiclone	Lunesta	☐		Y N		3 mg/d	Y N	*Y N	Y N	Y N U	1
Zolpidem	Ambien	☐		Y N		n/a	Y N	Y N	Y N	Y N U	0
Zaleplon	Sonata	☐		Y N		n/a	Y N	Y N	Y N	Y N U	0
**OTHER MEDICATIONS**											
Aripiprazole	Abilify	☐		Y N		n/a	Y N	Y N	Y N	Y N U	0
Olanzapine	Zyprexa	☐		Y N		n/a	Y N	Y N	Y N	Y N U	0
Quetiapine	Seroquel	☐		Y N		n/a	Y N	Y N	Y N	Y N U	0
Risperidone	Risperdal	☐		Y N		2 mg/d	Y N	Y N	Y N	Y N U	1
Prazosin	Minipress	☐		Y N		3 mg/d	Y N	Y N	Y N	Y N U	1
Lamotrigine	Lamictal	☐		Y N		n/a	Y N	Y N	Y N	Y N U	0
Levetiracetam	Keppra	☐		Y N		n/a	Y N	Y N	Y N	Y N U	0
Topiramate	Topamax	☐		Y N		100 mg/d	Y N	Y N	Y N	Y N U	1
Valproic Acid	Depakote	☐		Y N		n/a	Y N	Y N	Y N	Y N U	0
Gabapentin	Neurontin	☐		Y N		n/a	Y N	Y N	Y N	Y N U	0
Pregabalin	Lyrica	☐		Y N		n/a	Y N	Y N	Y N	Y N U	0
Carbamazepine	Tegretol	☐		Y N		n/a	Y N	Y N	Y N	Y N U	0
Clonidine	Catapres	☐		Y N		n/a	Y N	Y N	Y N	Y N U	0
Guanfacine	Tenex	☐		Y N		n/a	Y N	Y N	Y N	Y N U	0
Propranolol	Inderal	☐		Y N		n/a	Y N	Y N	Y N	Y N U	0
Atenolol	Tenormin	☐		Y N		n/a	Y N	Y N	Y N	Y N U	0
**UNLISTED (Write in below)**											
		☐		Y N		n/a	Y N	Y N	Y N	Y N U	0
		☐		Y N		n/a	Y N	Y N	Y N	Y N U	0

OFFICE USE ONLY

* When eszopiclone is used for augmentation, ≥3 weeks constitutes an adequate trial; 8 weeks is not required.

MEDICATION TOTAL POINTS: ______

PATIENT ID: **DATE:** **INTERVIEWER:**

E-TRIP PTSD PSYCHOTHERAPY TREATMENT RECORD

Have you ever received psychotherapy (talk therapy) for the treatment of PTSD? ☐ **No** ☐ **Yes**

If yes, please review the list of therapies below and check any you have received in the past or are receiving now. If you are unclear about the name of the therapy you have received, use the descriptions to identify which seems most like the treatment you received.

DO NOT WRITE IN THE GRAY-SHADED AREAS

PATIENT SECTION			OFFICE USE ONLY					
**Form of Psychotherapy**	**Check box if you received**		**Start date** MM/YYYY	**For PTSD?**	**No. of Sessions**	**Minimum No. of Sessions**	**If adequate trial, responded?**	**Points**
<u>TRAUMA-FOCUSED CBT</u>								
**Prolonged Exposure (PE):** You repeatedly went over the memory of the traumatic event by saying it out aloud with the therapist, and possibly by listening to a recording of you saying it while at home.	☐	OFFICE USE ONLY		Y N		6	Y N U	3
**Cognitive Processing Therapy (CPT):** You talked with the therapist about the “stuck points” that were the aspects of the event that were the most emotionally upsetting to you.	☐			Y N		6	Y N U	3
**Eye Movement Desensitization and Reprocessing (EMDR):** You went through the memory of the traumatic event while doing something repetitive, like following the therapist’s finger from side to side with your eyes.	☐			Y N		6	Y N U	2
**Trauma-Focused Cognitive-Behavioral Therapy (TFCBT):** You discussed thoughts about the trauma with the therapist and were assigned homework. You described the trauma aloud to the therapist.	☐			Y N		6	Y N U	3
**Narrative Exposure Therapy (NET):** You described the story of your life to the therapist and all emotions that went along with the events in your life.	☐			Y N		6	Y N U	2
**Written Exposure Therapy (WET):** You were asked to write a detailed account of your trauma in each session, emphasizing sounds and images as well as thoughts and emotions at the time of the trauma.	☐			Y N		5	Y N U	2
**Cognitive Restructuring:** You identified unhelpful beliefs you have and worked with the therapist to change those beliefs by examining the evidence for and against the beliefs.	☐			Y N		6	Y N U	0
**Stress Inoculation Therapy (SIT):** You learned coping skills to manage anxiety related to the trauma including tools to change unhelpful thoughts, relaxation exercises, and guided self-talk.	☐			Y N		6	Y N U	0
**Dialectical Behavior Therapy for PTSD:** You learned skills such as emotion regulation and distress tolerance and acceptance.	☐			Y N		6	Y N U	2
**Nightmare Imagery Rehearsal/CBT:** You were asked to write down a nightmare related to the trauma. You then altered the nightmare into a more positive story and rehearsed this repeatedly.	☐			Y N		6	Y N U	0
<u>INTERNET-BASED THERAPIES</u>								
**Internet-based Cognitive-Behavioral Therapy:** You used a website to communicate with a therapist and complete assignments. You wrote about your trauma and learned to share your experience with others.	☐			Y N		6	Y N U	3

TURN TO NEXT PAGE

E-TRIP PTSD PSYCHOTHERAPY TREATMENT RECORD

PATIENT SECTION		OFFICE USE ONLY					
Form of Psychotherapy	Check box if you received	Start date MM/YYYY	For PTSD?	No. of Sessions	Minimum No. of Sessions	If adequate trial, responded?	Points
**GROUP THERAPIES**							
**Group Interpersonal Psychotherapy:** In a group, you worked on problems with other people and PTSD such as arguments, social difficulties, changing roles, social isolation, and relationship triggers of PTSD symptoms.	☐		Y N		5	Y N U	3
**Cognitive-Behavioral Conjoint Therapy:** You and your partner went to therapy together. The therapist helped you understand how PTSD has affected your relationship and how to identify and prepare for triggers of PTSD symptoms, enhance communication with your partner, and approach rather than avoid difficulties.	☐		Y N		6	Y N U	2
**Group Cognitive-Behavioral Therapy:** In a group with a therapist, you wrote about your trauma and retold the details out loud at home. You may have learned mindfulness meditation, muscle relaxation, anger management, and role playing to improve social skills.	☐		Y N		6	Y N U	0
**COMPLEMENTARY AND ALTERNATIVE MEDICINE THERAPIES**							
**Mindfulness:** You worked on increasing your awareness and acceptance of the present moment, focusing on your physical sensations, emotions, and thoughts. You may have practiced yoga or meditation.	☐		Y N		6	Y N U	2
**Acupuncture:** Needles were placed into carefully chosen parts of your tissue or muscle and moved around by the practitioner.	☐		Y N		6	Y N U	2
**Healing Touch with Guided Imagery:** A trained practitioner performed this gentle noninvasive touch therapy to revitalize your energy and stimulate healing. You listened to a recording that helped you relax and change negative emotions about your trauma.	☐		Y N		6	Y N U	2
**OTHER THERAPIES**							
**Resiliency Intervention:** You learned about the concept of resilience. You developed skills such as paying attention to bodily sensations, cultivating positive emotions, and building social bonds, and used these skills when addressing your trauma.	☐		Y N		6	Y N U	2
**Present-Centered Therapy:** You learned skills to resolve problems you have in relationships that are caused by PTSD symptoms.	☐		Y N		6	Y N U	0
**Emotional Freedom Techniques:** You were asked to rate the distress level caused by each traumatic memory. You learned to tap your body at various energy centers while repeating positive affirmations about self-love and acceptance until the distress diminished.	☐		Y N		6	Y N U	2
**Mind-Body Bridging Program for sleep management:** You learned to relax your mind and body. You identified causes of your sleep difficulties.	☐		Y N		3	Y N U	2

OFFICE USE ONLY

TURN TO NEXT PAGE

E-TRIP PTSD PSYCHOTHERAPY TREATMENT RECORD

PATIENT SECTION		OFFICE USE ONLY					
**Form of Psychotherapy**	**Check box if you received**	**Start date** MM/YYYY	**For PTSD?**	**No. of Sessions**	**Minimum No. of Sessions**	**If adequate trial, responded?**	**Points**
**Supportive Therapy** (ex. Rogerian Therapy, Talk Therapy): You talked with your therapist about what was going on in your life and the kinds of stresses you were facing. The therapist encouraged and supported you and gave advice about how to manage problems.	☐		Y N		6	Y N U	0
**Relaxation:** You learned and practiced techniques to relax. These may include focusing on your breath, muscle relaxation, visualization, or the repetition of positive messages.	☐		Y N		6	Y N U	0
**<u>COMBINATION THERAPIES</u>**							
**Acupoint Stimulation added to Cognitive-Behavioral Therapy**: A needle was placed in a specific part of your body. A small electrical charge was sent through the needle.	☐		Y N		3	Y N U	1
**Skills Training in Affective and Interpersonal Regulation (STAIR):** You worked on social skills like being more aware, flexible and assertive. You learned techniques to regulate your emotions. You described your trauma in detail.	☐		Y N		6	Y N U	0
**Medication**: You were given one of the following medications just before or after the therapy session to make the therapy work better. Choose from list below.<br>• **D-Cycloserine (DCS)**	☐		Y N		n/a	Y N U	0
• **Methylene Blue**	☐		Y N		n/a	Y N U	0
• **Oxytocin**	☐		Y N		n/a	Y N U	0
• **Yohimbine**	☐		Y N		n/a	Y N U	0
• **Dexamethasone/Hydrocortisone/Prednisone**	☐		Y N		n/a	Y N U	0
• **MDMA (“Ecstasy”)**	☐		Y N		n/a	Y N U	0
• **Propranolol (Inderal)**	☐		Y N		n/a	Y N U	0

O F F I C E U S E O N L Y

PSYCHOTHERAPY TOTAL POINTS: ______

E-TRIP [EMORY TREATMENT RESISTANCE INTERVIEW FOR PTSD]

PATIENT: **DATE:** **INTERVIEWER:**

Hand the E-TRIP PTSD Medication Treatment Record and E-TRIP PTSD Psychotherapy Treatment Record to the patient.

INTERVIEW

1. Clarify date of onset of PTSD.
   ASK: ***When did your symptoms of PTSD begin?***
   Provide the patient with examples of PTSD symptoms if they are unsure.

2. Determine primary PTSD symptoms and impairments in functioning that led patient to seek treatment.
   ASK: ***What kinds of PTSD symptoms were you experiencing that caused you to seek treatment?***
   IF PATIENT IS UNCERTAIN, ASK: ***For example, did you have intrusive memories of the trauma, nightmares, or flashbacks?***
   - ***Did you make excessive efforts to avoid thinking about the event or avoid reminders of it?***
   - ***Did you feel distant from other people or lose interest in activities you once enjoyed?***
   - ***Did you have problems with sleep, concentration, or irritability?***

PROCEED THROUGH NUMBERS 3-5 FOR EACH MEDICATION TAKEN, ONE AT A TIME.
PROCEED THROUGH NUMBERS 6-8 FOR EACH PSYCHOTHERAPY RECEIVED, ONE AT A TIME.

PTSD MEDICATION TREATMENT RECORD

On the PTSD Medication Treatment Record, if the patient checked “No,” skip to Page 3 to assess psychotherapy treatments. If the patient checked “Yes,” continue below.

3. Confirm that the medication checked was taken after the date of onset of PTSD.

Start Date MM/YYYY — ASK: ***When did you start taking the*** *(MEDICATION NAME)****?*** Record in the E-TRIP PTSD Medication Treatment Record.

For PTSD? If the medication trial began **before** the onset of PTSD symptoms, circle “N” and **do not continue** to ask about the specific medication; if the medication trial began **after** the onset of PTSD symptoms, circle “Y” and **continue** to ask about the medication.

4. Determine if the medication treatment constituted an adequate trial. For each medicine taken for PTSD:

a. **Dose used** ASK: ***What was the highest dose of the*** *(MEDICATION NAME)* ***you took?*** Record in the E-TRIP PTSD Medication Treatment Record.

**Minimum effective dose**	If the dose reported is **less** than the minimally effective dose for that medication, **do not continue** to assess the specific medication. If it is **greater than or equal to** the minimally effective dose, **continue** to assess the medication.

b. Determine if the medication was used as monotherapy or as an augmentation ("add on") agent.

ASK: ***Was this medicine added on to a medicine you were already taking, so that you were taking both medicines on the same days?*** (If yes) ***Which medicine was it added on to?*** Record here. ____________

Used to augment?

- If the patient did not take the medication to augment another medication, circle "N."
- If the patient did take the medication to augment another medication, look up the point value for the medication you recorded in the above blank.
  - If the point value is **0 or 1 points or not listed**, do not continue to assess the augmentation medication; circle "N."
  - If the point value is **2 or 3 points**, the add-on medication is considered an augmentation agent, so circle "Y" under the Augment column and continue.

c. Determine the number of weeks that the patient took the medication.

≥8 weeks? ASK: ***For how many weeks in a row did you take that dose of*** *(MEDICATION NAME)****?*** Record in the E-TRIP PTSD Medication Treatment Record.

If the patient took dose for **<8 weeks,** circle N and **do not continue**. If the patient took dose for **≥ 8 weeks,** circle Y and **continue.**

NOTE: When eszopiclone is used for augmentation, ≥3 weeks constitutes an adequate trial; 8 weeks is not required.

d. Determine how many days the patient took the medication each week.

≥6 days/week? ASK: ***When you were taking that dose, did you take your*** *(MEDICATION NAME)* ***at least 6 days per week?***

Record in table: if the patient **did not** take the medication for at least 6 days per week, circle "N" and **do not continue** to assess this medication. If the patient **did** take the medication for at least 6 days per week, circle "Y" and **continue. This is an adequate trial.**

Patient responses should be confirmed using pharmacy prescription records or physicians' notes whenever such records are available.

5. **Responded? (Y, N, U)** Determine if the patient responded to each treatment. Response to a treatment is defined as *≥30% improvement in PTSD symptomatology*.

a. Assess Symptom Improvement. ASK: ***Which symptoms that you listed in the beginning of the interview*** (refer to patient's presenting problems on page 1, question 2) ***improved or did not improve?***

IF STILL UNSURE IF SYMPTOMS IMPROVED, ASK QUESTIONS BELOW:

- ***What did your family members or friends say about your response to the treatment?***
- ***Did you feel*** *(MEDICATION NAME)* ***made a clear difference in how you felt? Can you give me some examples of how it helped you?***

ASK: ***Considering all of these symptoms together, what percentage do you think your PTSD symptoms improved on this medication?***

RECORD:
- ☐ 0-29% improvement: non-response (N)
- ☐ 30-100% improvement: response (Y)
- ☐ Unsure of improvement (U)

NOTE: Patients who initially responded to a treatment, then lost response to the same treatment at a later time and never regained response should be scored as having a non-response to that treatment.

Points Code response on the E-TRIP PTSD medication treatment record. If the patient's PTSD symptoms improved 0-29% while taking the medication, circle "N." If the patient's PTSD symptoms improved 30-100% while taking the medication, circle "Y." If it is unclear if the patient's PTSD symptoms improved, circle "U." If you circled **"N," go to the "Points" column for that medication and circle the number listed.**

NOTE: A maximum of **TWO** failed SSRI treatment trials should be scored, so that the maximum number of points for failing to respond to SSRI medications is 6. Additional failed trials of SSRIs should not be scored.

REPEAT QUESTIONS 3-5 FOR EACH MEDICATION TAKEN.

After assessing all medication trials, add **total points** for nonresponse to adequate trials of medication treatments and **record** at the bottom of the E-TRIP PTSD Medication Treatment Record.

PTSD PSYCHOTHERAPY TREATMENT RECORD

On the E-TRIP PTSD Psychotherapy Treatment Record, if the patient checked "No," end the interview. If the patient checked "Yes," continue.

6. Confirm that the psychotherapy checked was started after the date of onset of PTSD.

Start Date MM/YYYY ASK: ***When did you begin treatment with*** *(PSYCHOTHERAPY NAME)****?*** Record in table.

For PTSD? If the psychotherapy began **before** the onset of PTSD symptoms, circle "N" and **do not continue** to ask about the specific psychotherapy; if the psychotherapy began **after** the onset of PTSD symptoms, circle "Y" and **continue** to ask about the psychotherapy.

If the patient checked multiple psychotherapies that occurred in a similar time frame, determine whether the separate therapies were actually conducted as part of a more comprehensive treatment package (e.g. "Relaxation" performed as part of prolonged exposure).

7. Determine if each treatment constituted an adequate trial.

ASK: ***How many sessions of*** *(PSYCHOTHERAPY NAME)* ***did you receive?*** Record in the E-TRIP PTSD Psychotherapy Treatment Record.

Minimum No. of Sessions	If the patient received **less** than the minimum number of sessions for that psychotherapy, **do not continue** to ask about the psychotherapy. If the patient received **greater than or equal** the minimum number of sessions, **continue** to ask about the psychotherapy. **This is an adequate trial.**

8. **Responded? (Y, N, U)** Determine if the patient responded to each treatment. Response to a treatment is defined as *≥30% improvement in PTSD symptomatology*.

a. Assess Symptom Improvement. (ASK) ***Which symptoms that you listed in the beginning of the interview*** (refer to patient's presenting problems on page 1, question 2) ***improved or did not improve?***

IF STILL UNSURE IF SYMPTOMS IMPROVED, ASK QUESTIONS BELOW:

- ***What did your family members or friends say about your response to the treatment?***
- ***Did you feel*** *(PSYCHOTHERAPY NAME)* ***made a clear difference in how you felt? Can you give me some examples of how it helped you?***

ASK: ***Considering all of these symptoms together, what percentage do you think your PTSD symptoms improved over the course of this psychotherapy?***

RECORD:

☐ 0-29% improvement: non-response (N)

☐ 30-100% improvement: response (Y)

☐ Unsure of improvement (U)

NOTE: Patients who initially responded to a treatment, then lost response to the same treatment at a later time and never regained response should be scored as having a non-response to that treatment.

Points

Code response on the E-TRIP PTSD Psychotherapy Treatment Record. If the patient's PTSD symptoms improved 0-29% over the course of the psychotherapy, circle "N." If the patient's PTSD symptoms improved 30-100% over the course of the psychotherapy, circle "Y." If it is unclear if the patient's PTSD symptoms improved, circle "U." If you circled **"N," go to the "Points" column for that medication and circle the number listed.**

REPEAT QUESTIONS 6-8 FOR EACH PSYCHOTHERAPY RECEIVED.
After assessing all psychotherapy trials, add **total points** for nonresponse to adequate trials of psychotherapy treatments and **record** at the bottom of the E-TRIP Psychotherapy Treatment Record.

POINTS: **MEDICATION _____**
PSYCHOTHERAPY _____
TOTAL _____
