# Supplementary material for: Assessing Treatment-Resistant Posttraumatic Stress Disorder: The Emory Treatment Resistance Interview for PTSD (E-TRIP)
Source: Behav Sci (Basel). 2014 Dec 8;4(4):511–27. doi: 10.3390/bs4040511 (PMC4287702; doi:10.3390/bs4040511)
Supplement: Supplementary File 2 [file behavsci-04-00511-s002.pdf]

E-TRIP [EMORY TREATMENT RESISTANCE INTERVIEW FOR PTSD]

E-TRIP SCORING SAMPLE

Ms. Stevens, a 34-year-old woman, met with her psychiatrist and divulged that her ex-husband, whom she divorced two years prior, had been extremely abusive to her for the majority of their five-year marriage. Since leaving him in March 2011, she has been experiencing daily intrusive memories and frequent nightmares of the abuse. She avoids driving past the neighborhood where they used to live, sometimes adding an hour to her driving time each day. She endorses a loss of interest in most activities that she used to enjoy and a sense of detachment from everyone in her life. She notes that she gets angry at people very easily, barely sleeps at night, and has difficulty concentrating.

Two months after leaving her husband, Ms. Stevens was diagnosed with posttraumatic stress disorder (PTSD) when she first saw a psychiatrist, who then prescribed her an SSRI antidepressant, fluoxetine (20 mg/day). She took the fluoxetine for 3 months, and then reported to her psychiatrist that she did not feel any better. Her psychiatrist switched her to a different SSRI antidepressant, sertraline (titrated to 100 mg/day), which she took for 5 months. When she saw no improvement with the sertraline, she sought out a new psychiatrist, who prescribed her two medications: escitalopram 20 mg/day and risperidone 2 mg/day. The psychiatrist explained that the risperidone would make the escitalopram work better. Ms. Smith reported that she rarely missed a dose of any of the medications she was prescribed. Throughout the two years, she saw a counselor for weekly supportive therapy through her church, but her PTSD symptoms still did not improve. When Ms. Smith presented at Emory, she explained that her symptoms were as bad as ever, despite being on the escitalopram and risperidone for over a year.

On the E-TRIP, Ms. Stevens' previous treatments would be marked as follows, with a total E-TRIP score of 7:

E-TRIP PTSD Medication Treatment Record

Generic name	Trade name	Check box if ever taken	Start date MM/YYYY	For PTSD?	Dose used	Minimum Effective dose	Used to augment?	≥ 8 weeks?	≥6 days/ week?	If adequate trial, responded?	Points†
Fluoxetine	Prozac	☑	*05/2011*	(Y) N	*20 mg/d*	20 mg/d	Y (N)	(Y) N	(Y) N	Y (N) U	(3)
Sertraline	Zoloft	☑	*08/2011*	(Y) N	*100 mg/d*	50 mg/d	Y (N)	(Y) N	(Y) N	Y (N) U	(3)
Escitalopram	Lexapro	☑	*01/2012*	(Y) N	*20 mg/d*	10 mg/d	Y (N)	(Y) N	(Y) N	Y (N) U	~~3~~
Risperidone	Risperdal	☑	*01/2011*	(Y) N	*2 mg/d*	2 mg/d	(Y) N	(Y) N	(Y) N	Y (N) U	(1)

MEDICATION TOTAL POINTS: ___*7*___

Only score nonresponse to a maximum of 2 adequate trials of SSRIs

E-TRIP PTSD Psychotherapy Treatment Record

Form of Psychotherapy	Check box if you received	Start date MM/YYYY	For PTSD?	No. of Sessions	Minimum No. of Sessions	If adequate trial, responded?	Points
**Supportive Therapy** (ex. Rogerian Therapy, Talk Therapy): You talked with your therapist about what was going on in your life and the kinds of stresses you were facing. The therapist encouraged and supported you and gave advice about how to manage problems.	☑	*05/2011*	(Y) N	*~100*	6	Y (N) U	(0)

PSYCHOTHERAPY TOTAL POINTS: ___*0*___

TOTAL: ___*7*___
